# Comparative analysis of endoscopic and histopathological findings in patients with chronic gastritis

**DOI:** 10.6026/973206300221160

**Published:** 2026-02-28

**Authors:** Mansi Rathore, Sakshi Chourasia, Purti Agrawal Saini, Yash Saxena

**Affiliations:** 1 Department of Pathology, L.N. Medical College & Research Centre, Bhopal, Madhya Pradesh, India; 2 Department of Pathology, Government Medical College, Satna, Madhya Pradesh, India; 3 Department of Pathology, Nandkumar Singh Chouhan Government Medical College, Khandwa, Madhya Pradesh, India

**Keywords:** Chronic gastritis, endoscopy, gastric biopsy, histopathology, mucosal patterns

## Abstract

Chronic gastritis is a common gastrointestinal disorder with variable clinical presentations and the accuracy of upper gastrointestinal endoscopy in reflecting histopathological changes remains inconsistent. Therefore, it is of interest to compare endoscopic findings with histopathological grading in 189 adult patients with chronic gastritis. The most frequent endoscopic finding was erythematous gastritis, followed by erosive and nodular changes, while histopathology revealed mild to severe chronic gastritis. Therefore, this study advances knowledge by providing valuable insights into the correlation between endoscopic and histopathological findings, which could improve diagnostic accuracy and management of chronic gastritis.

## Background:

Chronic gastritis is defined as a prolonged inflammatory condition of the gastric mucosa that may progress to atrophy, intestinal metaplasia and neoplastic transformation if left unrecognized or untreated. It remains one of the most frequently encountered pathologies in gastroenterology, with *Helicobacter pylori* infection recognized as the predominant etiological factor worldwide, affecting up to nearly half of the global population and contributing to mucosal inflammation and premalignant changes [1]. Histopathological examination of gastric mucosal biopsies is widely accepted as the diagnostic gold standard because it allows direct visualization and grading of cellular changes, including mononuclear infiltrate, glandular atrophy and intestinal metaplasia [2]. Upper gastrointestinal endoscopy is the frontline investigative modality for patients presenting with dyspeptic symptoms, providing visual assessment of mucosal integrity and the presence of erythema, erosions, atrophy or other gross abnormalities. Despite its crucial role in clinical evaluation, the predictive value of endoscopic findings for underlying histological severity is variable [3]. Several studies have reported notable discrepancies between endoscopic impressions and histological diagnoses, with findings ranging from low concordance in some cohorts to moderate agreement when standardized classification systems are applied [4]. Endoscopy may fail to detect microscopic inflammation in apparently normal mucosa, underscoring its limitations when used in isolation for the definitive diagnosis of chronic gastritis [5]. Given these considerations, correlating endoscopic and histopathological findings in patients with chronic gastritis are essential to optimize diagnostic accuracy and guide management [6]. Therefore, it is of interest to describe the correlation between endoscopic and histopathological findings in chronic gastritis, aiming to enhance diagnostic accuracy and clinical management.

## Materials and Methods:

## Study design and setting:

This retrospective observational study was conducted at a tertiary care teaching hospital. Medical records of patients who underwent upper gastrointestinal endoscopy for dyspeptic symptoms over a two-year study period were reviewed. Patient confidentiality was maintained throughout the data collection and analysis process. As this was a record-based study, informed consent was waived.

## Study population:

Patients aged 18 years and above who underwent diagnostic upper gastrointestinal endoscopy and were subsequently diagnosed with chronic gastritis based on histopathological examination of gastric biopsy specimens were considered for inclusion. The study included adults aged 18 years and above who underwent upper gastrointestinal endoscopy and had endoscopic findings suggestive of gastritis, with corresponding gastric biopsy reports confirming chronic gastritis. Only patients with complete medical records containing both endoscopic and histopathological documentation were considered. Patients were excluded if they had endoscopic or histological evidence of gastric malignancy, a history of gastric surgery, acute erosive or hemorrhagic gastritis, inadequate or poorly preserved biopsy specimens, or incomplete endoscopic or histopathological records.

## Sample size:

Based on the availability of eligible records during the study period and to ensure adequate representation for comparative analysis, a total of 189 patients fulfilling the inclusion criteria were included in the study. Consecutive sampling was employed to minimize selection bias. Relevant clinical and investigational data were extracted from hospital records using a structured data collection proforma.

The following variables were recorded:

[1] Demographic details (age, sex)

[2] Indication for endoscopy

[3] Endoscopic findings

[4] Histopathological diagnosis

Upper gastrointestinal endoscopy was performed using a standard flexible video endoscope. Endoscopic findings were documented by experienced endoscopists and categorized based on mucosal appearance, including erythema, nodularity, erosions, edema and mucosal atrophy. The severity of gastritis was classified as mild, moderate or severe based on the extent and intensity of mucosal changes observed during endoscopy. Gastric biopsy specimens were obtained from the antrum and/or body of the stomach during endoscopy. The tissues were fixed in formalin, processed routinely and stained with hematoxylin and eosin. Histopathological assessment was performed by qualified pathologists who were blinded to the endoscopic findings. Chronic gastritis was diagnosed based on the presence of mononuclear inflammatory cell infiltration in the lamina propria, with or without associated glandular atrophy or intestinal metaplasia. The severity of chronic inflammation was graded as mild, moderate, or severe. Endoscopic impressions were compared with histopathological findings to assess concordance. The diagnostic accuracy of endoscopy in detecting chronic gastritis was evaluated using histopathology as the reference standard. Data were entered into Microsoft Excel and analyzed using Statistical Package for Social Sciences (SPSS) software. Categorical variables were expressed as frequencies and percentages, while continuous variables were summarized as mean and standard deviation. The association between endoscopic and histopathological findings was assessed using the chi-square test. Agreement between endoscopic and histological diagnoses was evaluated using the kappa statistic. A p-value of less than 0.05 was considered statistically significant.

## Results:

A total of 189 patients with endoscopic suspicion of gastritis and available histopathological confirmation were included in the analysis. The demographic distribution of the study population is summarized in Table 1. The majority of patients were between 31 and 50 years of age, with a male predominance (59.3%). The spectrum of endoscopic findings observed during upper gastrointestinal endoscopy is detailed in Table 2. Erythematous gastritis was the most frequent endoscopic abnormality, identified in 86 patients (45.5%), followed by erosive gastritis in 41 patients (21.7%). Nodular and atrophic mucosal changes were noted in 28 (14.8%) and 22 (11.6%) patients, respectively. In 12 patients (6.4%), the gastric mucosa appeared normal despite clinical suspicion. Histopathological examination revealed varying degrees of chronic inflammation, as shown in Table 3. Moderate chronic gastritis was the most common histological diagnosis, observed in 81 patients (42.9%) and followed by mild chronic gastritis in 64 patients (33.9%). Severe chronic gastritis was identified in 44 patients (23.3%). A comparison between endoscopic severity and histopathological grading demonstrated a statistically significant association Table 4. Patients with mild endoscopic changes predominantly showed mild histological inflammation, whereas those with severe endoscopic findings were more likely to exhibit severe chronic gastritis on biopsy. The association was statistically significant (χ^2^ = 42.6, p < 0.001), with a kappa coefficient of 0.61, indicating substantial agreement between endoscopic and histopathological assessments. Further analysis assessing the relationship between specific endoscopic appearances and histopathological severity is presented in Table 5. Erosive and atrophic endoscopic patterns were significantly associated with moderate to severe chronic gastritis on histopathology, while erythematous changes showed a more heterogeneous distribution of histological severity. Notably, a subset of patients with endoscopically normal mucosa demonstrated histological evidence of chronic gastritis. The overall association between endoscopic appearance and histopathological severity was statistically significant (p < 0.01).

## Discussion:

In the present study involving 189 patients with chronic gastritis, a significant correlation between endoscopic severity and histopathological grading was observed (p < 0.01), with substantial agreement (kappa = 0.61). This aligns with evidence that endoscopic evaluation, while clinically useful, does not uniformly predict microscopic inflammation or morphological changes. Histopathological confirmation remains indispensable for accurate diagnosis and grading of gastritis [7]. Previous research highlights the limitations of endoscopy alone in diagnosing chronic gastritis. Some diagnostic studies have shown poor concordance between endoscopic impressions and histological findings, with notable discordance even when gross mucosal changes are apparent. For example, standard endoscopy demonstrated discordance with histology in a substantial proportion of patients, indicating that biopsies are essential for accurate diagnosis [8]. Such findings support our observation that histopathological assessment adds critical diagnostic depth beyond visual endoscopic evaluation. Moreover, studies indicate that normal endoscopic appearance does not exclude histological gastritis. A proportion of individuals with macroscopically normal mucosa have significant microscopic inflammation on biopsy, underscoring a key limitation of endoscopy as a standalone diagnostic tool [9]. Endoscopic sensitivity varies across lesion types and disease severity, with low sensitivity in some contexts such as intestinal metaplasia, further emphasizing the need for histological confirmation [10]. Although advanced imaging modalities, such as narrow-band imaging and magnifying endoscopy, have shown promise in enhancing predictive accuracy for specific histological features, conventional white-light endoscopy still requires routine biopsies to ensure accurate disease characterization, particularly for chronic inflammatory changes and premalignant lesions [11]. The clinical implication of these observations is clear: while endoscopy provides vital initial assessment and guides targeted biopsies, histopathological examination remains the gold standard for definitive diagnosis and severity grading of chronic gastritis. Integrating both modalities enhances diagnostic accuracy and informs optimal patient management.

## Conclusion:

We show a statistically significant association between endoscopic findings and histopathological severity in chronic gastritis, though the correlation was not consistent across all mucosal patterns. Erosive and atrophic changes were more reliably linked with moderate-to-severe inflammation, whereas erythematous mucosa showed wide histological variability. Chronic gastritis was also detected in some patients with normal endoscopy, reinforcing the need for routine gastric biopsy alongside endoscopic assessment.

## Figures and Tables

**Table 1:** Demographic profile of the study population (n = 189)

**Variable**	**Frequency**	**Percentage (%)**
Age group (years)		
18-30	34	18
31-40	48	25.4
41-50	46	24.3
51-60	37	19.6
>60	24	12.7
Sex		
Male	112	59.3
Female	77	40.7

**Table 2:** Distribution of endoscopic findings (n = 189)

**Endoscopic finding**	**Number**	**Percentage (%)**
Erythematous gastritis	86	45.5
Erosive gastritis	41	21.7
Nodular gastritis	28	14.8
Atrophic changes	22	11.6
Normal-appearing mucosa	12	6.4

**Table 3:** Histopathological severity of chronic gastritis (n = 189)

**Histopathological grade**	**Frequency**	**Percentage (%)**
Mild chronic gastritis	64	33.9
Moderate chronic gastritis	81	42.9
Severe chronic gastritis	44	23.3

**Table 4:** correlation between endoscopic and histopathological severity (n = 189)

**Endoscopic severity**	**Mild (HPE)**	**Moderate (HPE)**	**Severe (HPE)**	**Total**	**P Value**
Mild	46	24	8	78	<0.01
Moderate	14	41	14	69	
Severe	4	16	22	42	
Total	64	81	44	189	

**Table 5:** Association between endoscopic appearance and severity of chronic gastritis on histopathology (n = 189)

**Endoscopic finding**	**Mild HPE**	**Moderate-Severe HPE**	**Total**	**p-value**
Erythematous	38	48	86	<0.001
Erosive	9	32	41	
Nodular	7	21	28	
Atrophic	4	18	22	
Normal mucosa	6	6	12	
Total	64	125	189	
